# Microbial cell factories based on filamentous bacteria, yeasts, and fungi

**DOI:** 10.1186/s12934-023-02025-1

**Published:** 2023-01-30

**Authors:** Qiang Ding, Chao Ye

**Affiliations:** 1 School of Life Sciences, Anhui University, Hefei, 230601 China; 2 School of Food Science and Pharmaceutical Engineering, Nanjing Normal University, Nanjing, 210023 China; 3 Key Laboratory of Human Microenvironment and Precision Medicine of Anhui Higher Education Institutes, Anhui University, Hefei, 230601 Anhui China; 4 Anhui Key Laboratory of Modern Biomanufacturing, Hefei, 230601 Anhui China

**Keywords:** Filamentous microorganisms, Cellular tolerance, Metabolic engineering, Screening, Microbial cell factories

## Abstract

**Background:**

Advanced DNA synthesis, biosensor assembly, and genetic circuit development in synthetic biology and metabolic engineering have reinforced the application of filamentous bacteria, yeasts, and fungi as promising chassis cells for chemical production, but their industrial application remains a major challenge that needs to be solved.

**Results:**

As important chassis strains, filamentous microorganisms can synthesize important enzymes, chemicals, and niche pharmaceutical products through microbial fermentation. With the aid of metabolic engineering and synthetic biology, filamentous bacteria, yeasts, and fungi can be developed into efficient microbial cell factories through genome engineering, pathway engineering, tolerance engineering, and microbial engineering. Mutant screening and metabolic engineering can be used in filamentous bacteria, filamentous yeasts (*Candida glabrata, Candida utilis*), and filamentous fungi (*Aspergillus* sp., *Rhizopus* sp.) to greatly increase their capacity for chemical production. This review highlights the potential of using biotechnology to further develop filamentous bacteria, yeasts, and fungi as alternative chassis strains.

**Conclusions:**

In this review, we recapitulate the recent progress in the application of filamentous bacteria, yeasts, and fungi as microbial cell factories. Furthermore, emphasis on metabolic engineering strategies involved in cellular tolerance, metabolic engineering, and screening are discussed. Finally, we offer an outlook on advanced techniques for the engineering of filamentous bacteria, yeasts, and fungi.

## Background

Microbial cell factories provide an environmentally friendly strategy to produce industrial chemicals, which include food additives, pharmaceutical intermediates, monomers of bio-based materials, dietary amino acids, and four-carbon organic acids [1–3]. The optimal chassis can achieve the maximal efficiency of chemical production, while filamentous bacteria, yeasts, and fungi possesses excellent protein secretion ability, and can be fermented on low-cost materials [4–6]. Thus, to improve the production performance of filamentous bacteria, yeasts, and fungi, numerous strategies were developed, such as genome engineering, promoter engineering, biosensor engineering, compartment engineering, and quorum sensing systems [7–11].

**Fig. 1:**
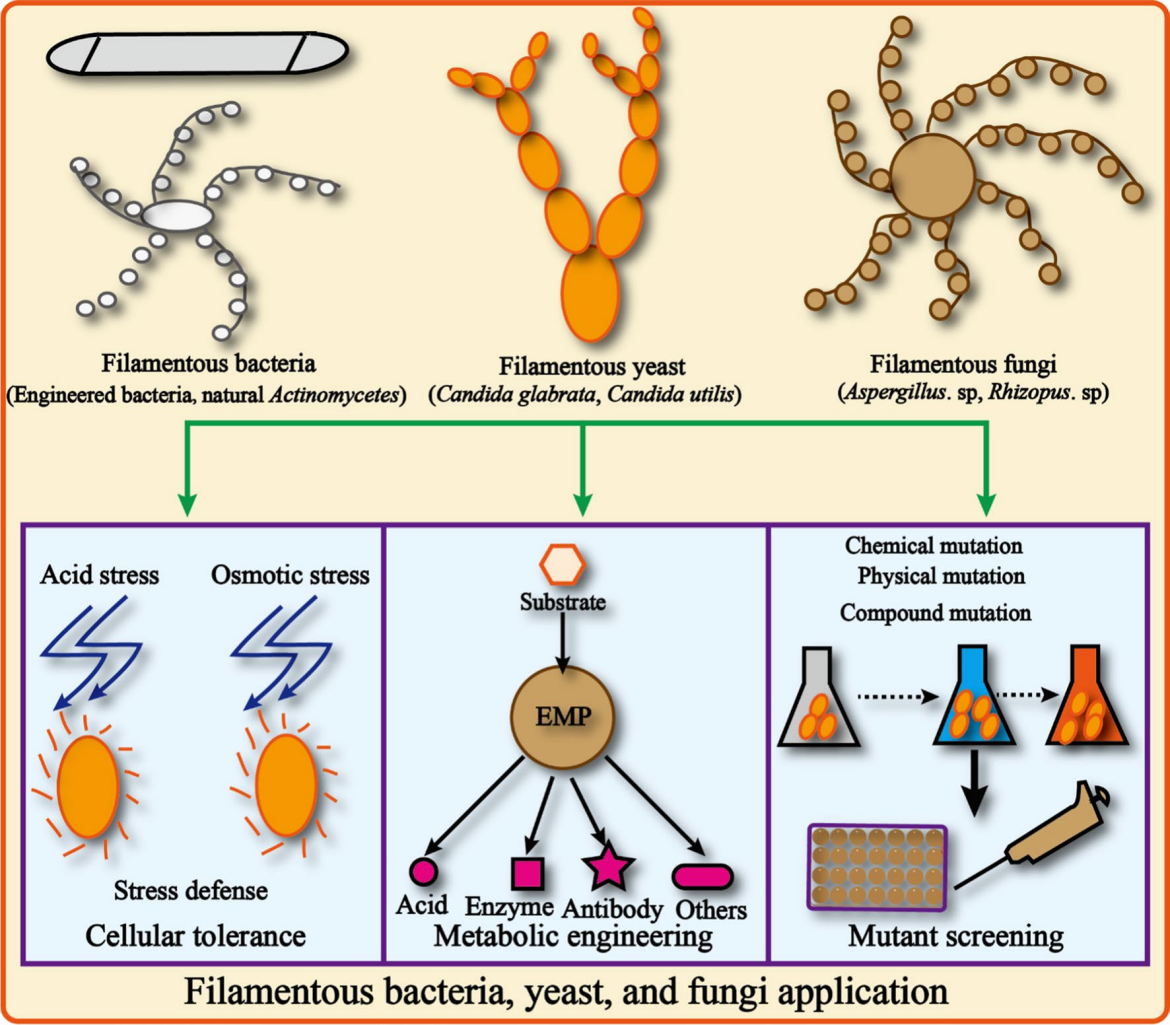
Filamentous bacteria, yeast, and fungi application in microbial cell factory. The main filamentous microbiology were the *filamentous bacteria (*Engineered bacteria, natural *Actinomycetes), filamentous yeast (Candida glabrata, Candida utilis), and filamentous fungi (Aspergillus. sp, Rhizopus oryzae),* which were the important industrial strains for chemical production. The review hightlight the cellular tolerance, metabolic engineering, and mutant screening application in the filamentous application

Although significant breakthroughs were achieved, it is still necessary to understand the physiological mechanisms for expanding the production potential of filamentous bacteria, yeasts, and fungi [12–14]. In this review, we comprehensively summarized the cell tolerance, mutant screening, and metabolic engineering applications in filamentous bacteria (*Escherichia coli, Corynebacterium glutamicum, Pseudomonas mendocina, Actinomycetes*), filamentous yeasts (*Candida glabrata, Candida utilis*), and filamentous fungi (*Aspergillus* sp., *Rhizopus oryzae*), and highlighting that bacteria, yeasts, and fungi are a promising chassis for the engineering of filamentous cells (Fig. 1).

## Application of filamentous bacteria as microbial cell factories

Filamentous bacteria mainly include the engineered filamentous bacteria and natural Actinomycetes. The cell morphology can be regulated into a filamentous type for increasing the synthesis of polyhydroxyalkanoates, enzymes, alginate oligosaccharides and other natural products (Table 1).

**Table 1:** Metabolic engineering application in filamentous bacteria

Category	Strategies	Strains	Products	Yield (g/g)	Titer (g/L)	Productivity (g/L/h)	Refs.
Filamentous bacteria	Engineered bacteria	*E. coli ftsZ* (1 + 2 + 3 + 5)	PHB	–	6.39	–	[17]
		*E. coli* BL21 (DE3) (pet28asulA, p15apCAB)	P(3HB)	–	2.27	–	[18]
		*Halomonas* TD08	PHB		82.04% wt	–	[19]
		*C. glutamicum *AP4	5-aminolevulinic acid	–	2.53	0.07	[20]
		*E. coli ftsW*1 + *ftsW*4	PHB	–	93% wt	–	[21]
	Natural *Actinomycetes*	*E. coli* PLH4	PLH		52% wt	–	[22]
		*S. spinosa*	Spinosad	–	0.075	–	[33]
		*S. coelicolor*	Pristinamycin II	–	2.2	0.018	[25]
		*S. hygroscopicus-*Δk-DR	Rapamycin	–	0.25	0.002	[139]
		*S. pristinaespiralis* ∆*papR5* + *R4R6*/BAC-F1F15	Pristinamycin II	–	1.16	0.009	[36]
		*Streptomyces* sp. RM7011	FK506	–	0.16	–	[23]
		*S. venezuelae*	Tylactone		0.006	–	[37]
		*S. coelicolor*	Rapamycin	–	1.83	0.007	[7]

### Application of engineered bacteria as microbial cell factories

Metabolic engineering in engineered filamentous bacteria (*Escherichia coli, Corynebacterium glutamicum, Pseudomonas mendocina*) mainly involves in cellular divisome, cell wall, and lifespan. The cellular divisome is a protein complex that promotes the formation of Z ring at the division site, which is recognized as the starting point of cell division, and its core protein FtsZ is an important gene for forming filamentous bacteria. Therefore, the *ftsZ* gene can be repressed, the *sulA* gene can be overexpressed, and the *minCD* genes can be overexpressed to disrupt the divisome assembly, which can produce filamentous bacteria [15, 16]. For example, the CRISPRi technique was utilized to repress the *ftsZ* gene in the *E. coli* chromosome, which resulted in filamentous *E. coli*. Then, the biopolyester polyhydroxybutyrate (PHB) pathway was overexpressed to form the *E. coli ftsZ* (1 + 2 + 3 + 5), in which the PHB content and CDW were increased to 61.17% and 10.44 g/L, respectively [17]. Furthermore, the *sulA* gene was overexpressed to construct the *E. coli* BL21 (DE3) (pet28asulA, p15apCAB) which is also a filamentous *E. coli*. As a consequence, the P(3HB) content and CDW of *E. coli* BL21 (DE3) (pet28asulA, p15apCAB) reached 26.54% and 8.54 g/L, respectively [18]. In addition, the *minCD* genes can also be used to increase the cell volume, and *Halomonas* TD08 (pSEVA341-MinCD)-24 as able to accumulate 82.04% wt PHB [19]. The cell wall as the main outer barrier can be weakened to enhance the cellular permeability and elasticity to accumulate more chemicals. The *pbp2b* gene encoding a penicillin-binding protein was deleted in *C. glutamicum* to improve the cellular permeability and length. As a result, the 5-aminolevulinic acid titer of *C. glutamicum *AP4 was increased to 2.53 g/L, representing a 22% improvement [20]. Another example is to regulate the cellular elasticity to form filamentous bacteria. For example, the *ftsW* gene in the cell wall synthesis pathway was repressed to weaken the cell rigidity and produce longer cells. For example, the engineered *E. coli ftsW1* + *ftsW4* (pBHR68) which can produce the 93% wt PHB [21] was built using the CRISPRi system. The replicative lifespan is the number of daughter cells produced before aging, which can be manipulated to form the filamentous type. For example, two fluorescence proteins were utilized to locate the old pole and new pole in *E. coli*. Furthermore, a recombinase A118-based logic gate was constructed to dynamical regulate the replicative lifespan and poly(lactate-co-3-hydroxybutyrate) pathway. Finally, the daughter (rejuvenated cell) and mother (storage cell) were formed in *E. coli* PLH4, which can accumulate 52% wt poly(lactate-co-3-hydroxybutyrate) [22].

### Application of natural *Actinomycetes* as microbial cell factories

*Actinomycetes* are a special group of prokaryotes that can form a branching mycelium with conidia, and grow in a mycelium-like shape, mainly to reproduce by spores [8, 11, 23, 24]. Recently, genome mining approaches and gene expression tools have made it possible to access the unexploited potential of *Actinomycetes* to produce novel natural products [11, 25, 26]. Thus, the mutant screening and metabolic engineering can be utilized to engineer *Actinomycetes* for enhancing the synthesis of antibiotics and other drugs (Table 1).

#### Mutant screening in* Actinomycetes*

Mutant screening is an efficient strategy to enhance the production of chemicals in *Actinomycetes,* which mainly includes physical, chemical, and iterative mutagenesis [27]. ARTP, ^60^Co-γ, and UV irradiation are typical strategies for physical mutagenesis, causing changes in molecular structure and altering production performance [28]. For example, ultraviolet (UV) radiation, ethidium bromide (EB), and ethyl methanesulfonate (EMS) were used to screen mutants of *Streptomyces avermitilis* 41445 with increased production of avermectin B1b. The UV 45 (3) strain could synthesize 254.14 mg/L of avermectin B1b, representing a 14.95-fold increase over the parental strain [29]. Chemical mutagenesis, mainly uses EMS, DES, and NTG, which can cause gene mutation, and chromosome breaks. Notably, EMS mutagenesis resulted in higher thrombinase activity than UV mutagenesis in *Streptomyces*. The maximum specific growth rate and inhibition constant of the mutant was increased by 45.94% and 17.24%, to 0.5457 /h and 155.1 mg/mL, respectively [30]. Iterative mutagenesis has a synergistic effect, and the rational combination of two or more mutagenesis methods has a better effect than single mutagenesis [31]. For example, the chemical mutagen N-methyl-N-nitroso-N’-nitroguanidine (NTG) and physical mutagenesis with ultraviolet (UV) irradiation were used as iterative agents to screen *Streptomyces* mutant*s.* The best mutant N3 was then further optimized to improve the yield of amphotericin B. The final yield reached 5260 mg/L, representing a 906.9% improvement over the control strain ZJB 20130827 [31].

#### Metabolic engineering applications in* Actinomycetes*

Genome engineering, pathway engineering, and genetic circuits can improve the efficiency of microbial cell factories with the aid of metabolic engineering strategies and synthetic biology tools (Table 1) [11]. Genome engineering is based on multi-omics data and genome-wide metabolic models, which can reprogram the metabolic network at the system level [8]. Multi-omics data are needed to understand the regulatory network and metabolic mechanism of a production strain. For example, the transcriptome and proteome are used to describe the synthesis of model antibiotics in *Streptomyces coelicolor*. A total of 3570 transcriptions start sites and 230 small RNAs were identified and determined to assist the antibiotic discovery and development [32]. In the application of genome-scale metabolic models, the metabolic network reconstruction was used to improve spinosad production. Amino acid supplementation requirements, transhydrogenase regulation, and target genes were identified based on the in silico metabolic network models. Finally, the spinosad titer was improved to 75.32 mg/L, representing an 86% increase compared to the control [33]. Another example is the use of multiplexed site-specific genome engineering for enhancing the pristinamycin II titer to 2.2 g/L in shake flasks [25].

In addition to pathway engineering, metabolic engineering must also account for the precursor supply [9, 10]. An inducible expression system and auto-inducible expression system can be used to maximize the precursor production in streptomycetes [34]*.* For example, the oxytetracycline responsive repressor OtrR, and its operator were used to construct a concentration-dependent genetic circuit. When oxytetracycline was added in the range of 0.01–4 μM, it induced significant GFP expression, providing valuable potential regulatory elements for streptomycetes [35]. In pathway optimization, promoter engineering, ribosome binding sites, and terminators can be utilized to enhance the pathway flux and deleted byproduct [24]. For example, the methylmalonyl-CoA formation pathway was overexpressed to regulate the PCC pathway, which increased the FK506 titer to 164.92 mg/L, representing a 75% improvement [23]. A similar approach was used to optimize the pristinamycin II (PII) gene cluster in *S. pristinaespiralis*, which increased the product titer to 1.16 g/L in the 5-L fermenter, representing a 5.26-fold enhancement [36]. Another example is to eliminate the byproduct pathway of ethylmalonyl-CoA in *S. venezuelae*, which increased the product titer to 5.5 mg/L, representing a tenfold increase [37]. In addition to high-throughput screening, metabolic engineering can also be applied in *Actinomycetes* [11]. For example, a low dosage of ketoconazole was utilized to isolate the antifungal activity from more than 20,000 extracts [38].

Moreover, the intelligent switches and genomic information could help the development of specific genetic circuits in *Actinomycetes*, which mainly include quorum sensing systems, biosensor engineering, promoter libraries, and RNA interference. Quorum sensing systems are the most widely used dynamic regulation tools in industrial microorganisms, which can optimize the cellular metabolism and product synthesis. Therefore, the quorum sensing system was also developed in the *S. rapamycinicus*, and a CRISPRi system was also integrated with this system to construct the EQCi system for rapamycin production. Finally, the three key nodes were downregulated to channel the carbon flux toward rapamycin synthesis via fine-tuned repression through this *ermEp*-driving EQCi circuit. The highest product titer reached 1.836 g/L, representing a 660% improvement [7]. For biosensor engineering, the microfluidic platform and biosensor engineering were combined to screen erythromycin-producing strains of *Saccharopolyspora erythraea*, and a 50% improvement of the erythromycin yield was obtained [39]. In addition, promoter libraries can assist the optimization of genetic circuit. For example, the − 10 and − 35 consensus sequences of the ermEp1 promoter were changed in *Actinomycetes*, resulting in 2% to 319% relative changes of the expression strength [26]. In addition, the antisense RNA interference strategy was utilized to downregulate the expression of UDGs, resulting in a 2.8- to 65.8-fold improvement of editing efficiency in *S. lividans* 6 [40].

## Application of filamentous yeasts as microbial cell factories

*Candida albicans, Candida tropicalis, Candida parapsilosis, Candida dubliniensis, Candida glabrata* and *Candida utilis* are typical yeasts that grow in the filamentous form, which can form the blastospore and pseudohyphae. Among them, *Candida glabrata* and *Candida utilis* possess potential industrial application value for the production of high-value proteins, enzymes, and organic acids (Table 2).

**Table 2:** Metabolic engineering application in filamentous yeast

Category	Strategies	Strains	Products	Yield (g/g)	Titer (g/L)	Productivity (g/L/h)	Refs.
Filamentous yeast	*C. glabrata*	*T. glabrata* CCTCC M202019	Pyruvate	0.44	40.2	0.56	[55]
		*T. glabrata* T.G-PMS	L-malate	–	8.5	0.12	[56]
		*T. glabrata* T.G(4ade12)-PMS-P160A	Fumarate	0.15	9.2	0.15	[44]
		*T. glabrata* TGFA091-16	Fumarate	0.13	33.13	0.35	[58]
		*T. glabrata* T.G-4G-S	Fumarate	0.09	8.51	0.12	[59]
		*C. glabrata* CGhif-6	Pyruvate	–	53.1	0.74	[60]
		*T. glabrata* T.G-4G-S(1:1:2)-P(M)-F(H)	Fumarate	–	21.6	0.3	[59]
		*C. glabrata* C-Δadh-Δald-Δbdh -ScPDC1-NOX	Acetoin		7.33	0.11	[42]
		*C. glabrata* Cgcrz1 ∆ /CgCRZ1	Pyruvate	–	1.6-fold	1.6-fold	[47]
		*C. glabrata* HTUΔ/CgMED15B	Pyruvate		1.61-fold	1.61-fold	[140]
		*T. glabrata* T. G-KS(H)-S(M)–A-2 S	Fumarate	–	15.7	0.22	[62]
		*C. glabrata* CmA5	Acetoin	–	3.26	0.05	[61]
		*C. glabrata* CGΔura3-SpMPC1	Pyruvate	–	2.34-fold	2.34-fold	[63]
		*T. glabrata* CCTCC M202019	Pyruvate	0.44	40.2	0.56	[55]
		*T. glabrata* T.G-PMS	L-malate	–	8.5	0.12	[56]
	*C. utilis*	*C. utilis*	Glutathione	–	0.33	–	[70]
		*C. utilis* WSH 02–08	Glutathione	–	1.33	–	[67]
		*C. utilis*	Lipase activity	–	0.6 (A450)	–	[74]
		*C. utilis*	Biotin	–	1.8 mg/L	–	[72]
		*C. utilis* TMS411	Isopropanol	0.14	27.2	0.24	[75]
		*C. utilis* WSH 02–08	Glutathione	–	0.74	–	[68]
		*C. utilis* TMS174	Ethanol	–	17.4	0.58	[76]
		Cupdc1-null mutant	Lactate	0.95	103.3	3.44	[141]

### Applications of* Candida glabrata* as microbial cell factories

Although *C. glabrata* is potentially pathogenic, it can effectively produce organic acids in industrial application [41–43]. In addition, *C. glabrata* has facilitated the cost-effective biotechnological production of various chemicals and materials based on cellular tolerance and metabolic engineering. Further improvement of cellular tolerance, mutant screening, and metabolic engineering can efficiently reinforce the efficiency of microbial cell factories.

#### Cellular tolerance in* Candida glabrata*

Tolerance to acid and osmotic stress are the main cellular tolerance traits in the industrial strains of *C. glabrata*, which can improve the efficiency of microbial cell factories [44, 45]. The tolerance to HCl and l-malate have been identified as important acid-tolerance traits in *C. glabrata* [46]*.* For HCl stress, the CgCrz1p transcription factor was deleted and overexpressed to identify the necessity of growth in a pH 2.0 environment. It was found that membrane composition may be regulated by CgCrz1p transcription factor through global transcriptome analysis. In the Cg*crz1*/CgCRZ1 strains, the membrane integrity was decreased by 35.1% according to PI staining and membrane fluidity was increased by 13% according to anisotropy values [47]. Similarly, the CgMed16 was knocked out and overexpressed to confirm the importance of maintaining survival under the high titer l-malate stress. Furthermore, the membrane integrity was maintained by regulating CgMed16 and its corresponding transcription factors *CgYAP3* and *CgUSV1* based on transcriptome analysis. Finally, a dynamic tolerance system was constructed to improve the l-malate titer to 35.5 g/L, representing a 32.5% improvement [48]. Osmotic stress is mainly caused by high salt concentrations in *C. glabrata.* In a study on stress resistance to 1.5 M NaCl, the interaction of *CgHog1* with *CgRds2* was confirmed through transcriptome analysis to regulate glycerophospholipid metabolism. Finally, the membrane integrity and cell growth were increased by 12.1% and 10.2%, respectively [49]. In addition, adaptive laboratory evolution can be used to enhance salt tolerance. For example, the *S. cerevisiae* mutant XCG001 was obtained through adaptive laboratory evolution to growth in the presence of 1.5 M NaCl for 80 days. Further transcriptomic analysis revealed that the *ELO2* gene is related to osmotic stress and it was overexpressed to improve the membrane integrity the *S. cerevisiae* mutant XCG010. Finally, the membrane integrity, cell growth, and cell survival of *S. cerevisiae* were improved by 24.4, 21.9, and 22.1%, respectively [50].

#### Mutant screening in* Candida glabrata*

Mutation breeding can enhance the pathway efficiency to improve the production performance *of C. glabrata*. For pathway efficiency, cofactor availability, glycolysis flux, and cell tolerance can be increased through pathway regulation based on mutation breeding [51, 52]. The cofactor availability is mainly related to energy metabolism and cofactor supply. The energy metabolism is mainly focused on ATP utilization, which can increase the production of ATP-dependent chemicals. For example, neomycin was used to screen mutants resistant to inhibition of F_0_F_1_-ATPase. Thus, a neomycin-resistant mutant N07 was obtained to decrease the F_0_F_1_-ATPase and ATP levels by 65% and 24%. Finally, the 42.9% and 34% enhancements in pyruvate productivity and glucose consumption were achieved, respectively [51]. In addition, an ethanol-utilization and respiratory-deficient screening strategy can also be used to redistribute cofactors. For example, the ethanol-utilizing mutant WSH-13 was obtained to enhance the alcohol dehydrogenase activity by 110%. As a result, the NADH/NAD^+^ ratio was decreased to 0.22, while the glucose consumption rate and pyruvate production were increased by 26.3% and 22.5%, respectively [52, 53]. In addition, ethidium bromide was used to screen respiratory-deficient mutants. As a result, the specific enzyme activity of complex I, complex I + III, complex II + III, and complex IV in mutant RD-18 were decreased to 2.5, 17.8, 26.2, and 14.4 U/mg protein, respectively, resulting in the production of 43.8 g/L pyruvate and consumption of 97.9 g/L glucose [53]. Similarly, a respiratory-deficient mutant was selected through ethidium bromide mutagenesis, due to which the electron transfer chain activity was reduced. Finally, the mutant strain N07 exhibited increases of the pyruvate titer and glucose consumption by 33.4% and 46.9%, respectively [54].

#### Metabolic pathway engineering in* Candida glabrata*

Metabolic engineering applications mainly include optimization of energy metabolism, pathway construction, and compartment engineering to increase the efficiency of microbial cell factories (Table 2) [44]. To optimize energy metabolism, the energy utilization was enhanced and the ATP futile cycle was regulated to enhance pyruvate production in *C. glabrata.* For example, citrate can be added to resist tolerance by promoting energy metabolism, so that the ∆pH can be maintained with a higher ATP level. Finally, the pyruvate titer was increased by 28% and 32.5% at pH 4.5 and 5.0, respectively [45]. Another example is to reduce the ATP waste. The ATP futile cycle system was constructed for regulating the central metabolism to automatically reduce the intracellular ATP content. Thus, the ATP-FCS was optimized and coordinated with other metabolic pathways to increase pyruvate synthesis. Finally, the maximum pyruvate titer, specific production efficiency, and substrate conversion rate of pyruvate were respectively increased by 33.1%, 55.0%, and 74.2% [55].

Novel pathways can be introduced to produce valuable chemicals in *C. glabrata.* For example, as a pyruvate-producing strain, *C. glabrata* can be used as a platform to produce l-malate based on the pyruvate pool. To achieve this, the RoMDH and RoPYC enzymes from *Rhizopus oryzae* NRRL1526 and RoMAE1 *Schizosaccharomyces pombe* ATCC 26189 were overexpressed from a plasmid to form the T.G-PMS strain. Finally, 8.5 g/L l-malate was obtained, representing a tenfold improvement over the starting strain [56]. Another example is to engineer the urea cycle and purine nucleotide cycle for fumarate production. The engineered strain T.G-ASL(H)-ADSL(L)-SpMAE1 produces the 8.83 g/L fumarate, representing a 67.9-fold increase over the starting strain T.G-212 [57]. Moreover, to enhance the chemical production, modular pathway engineering, scaffold engineering, and oxygen-inducible promoters can enhance pathway efficiency. Modular pathway engineering was utilized to assemble the enzymes as the whole regulation target to optimize the expression strength. For example, the PMFM module, KSSS module, and RPSF module were separated in fumarate production. By combining the synthesized DNA-guided scaffolds and designed sRNA switch, the fumarate titer was increased to 33.13 g/L with a yield of 0.33 g/g [58]. Scaffold engineering can also enhance fumarate production. When a DNA scaffold was used for modular control over synthetic pathways for fumarate production, the engineered strain T.G-4G-S produced 8.51 g/L fumarate with a yield of 0.09 g/g [59]. An oxygen-inducible expression strategy can be established through hypoxia-inducible factor 1 to increase the relative pathway enzyme activity for pyruvate production at the low DO level. Using this strategy, the pyruvate titer reached 53.1 g/L with a 10% DO level in the 5-L fermenter [60].

Compartment engineering and carrier engineering can concentrate the pathway enzymes for increasing the transport of intermediates. Compartment engineering can be utilized to produce acetoin based on the precursor pyruvate. For example, the mALS and mALDC enzymes of the heterologous acetoin pathway were targeted into the mitochondria of *C. glabrata* to increase the relative enzyme concentration. Finally, 3.26 g/L acetoin was obtained in the strain CmA5, representing a 59.8% improvement compared to the strain without mitochondrial targeting [61]. In another example, the fumarate pathway as targeted to the mitochondria to obtain a 15.76 g/L fumarate yield [62]. Carrier engineering was also used to enhance the mitochondrial pyruvate carrier to transport pyruvate into the mitochondria. Furthermore, plasma membrane expression of MPCs decreases the intracellular pyruvate content. Finally, 3.38- and 3.47-fold increases of the maximum specific growth rate and specific pyruvate production rate were obtained in the engineered strain CGΔura3Sp-MPC2 [63].

### Application of *Candida utilis* as microbial cell factories

*Candida utilis* can utilize five- or six-carbon sugars, and does not produce alcohol under aerobic conditions. Moreover, it can not only use the waste liquid of the sulfite process, but also use molasses and wood hydrolysate to produce proteins [64, 65]. Further optimization of cellular tolerance and metabolic engineering can assist the development of *Candida utilis* cell factories.

#### Cellular tolerance in* Candida utilis*

Acid stress and oxidative stress can be applied to enhance the production of chemicals in *Candida utilis.* Glutathione (GSH) production in *C. utilis* is increased as defense against acid stress through regulating the intracellular ATP ratio and pH [66–68]. In another interesting example, transcriptomic and RNA-Seq data were used to identify the molecular mechanism of the acid stress response in *C. utilis.* The upregulated genes were mainly involved in intracellular ATP supply and metabolism of sulfur-containing substances, which could improve the glutathione production and intracellular organic selenium [69]. For oxidative stress, the activities of catalase (CAT) and GSH reductase (GR) can be enhanced by H_2_O_2_ due to the stress-induced adaptive responses in *C. utilis*. Therefore, different concentrations and times of H_2_O_2_ addition were used to improve the glutathione production, which increased the DCW and glutathione titer to 14.24 g/L and 328.4 mg/L in the 7-L fermenter, respectively [70].

#### Metabolic engineering applications in* Candida utilis*

Genetic tools, enzyme expression and pathway construction are the main metabolic engineering strategies in *C. utilis.* In addition to genetic tools, the gene assembly and gene disruption are generally used for genetic manipulation, while the Cre-loxP system for gene disruption is a vital tool to assist the development of *C. utilis* cell factories [71]. For example, CuURA3 disruption can be achieved using this system, which is a practical recombinant DNA tool [71]. Engineering strategies for enzyme expression mainly include protein expression and protein secretion [72, 73]. For example, the *Candida antarctica* lipase B was fused to *C. utilis* Gas1 cell wall protein, which can be used for surface display. As a result, the lipase activity (A405) was increased by 60% [74]. In a different approach, the BIO2 gene was integrated into the chromosome of *C. utilis* to produce 1.9 mg/L of biotin [72]. In addition, ethanol and isopropanol synthesis pathways were constructed in *C. utilis* [75, 76]. For example, when the *ctfA, ctfB, adc* and *sadh* genes were overexpressed in *C. utilis*, it produced 0.21 g/L isopropanol. Furthermore, the ACS2 and ERG10 genes were expressed to enhance the availability of acetyl-CoA, and *C. utilis* TMS411 produced the 9.52 g/L isopropanol in shake flasks, as well as and 27.2 g/L in the bioreactor [75].

## Applications of filamentous fungi as microbial cell factories

*Aspergillus*, *Rhizopus*, *Penicillium*, and *Mucor* are important industrial microorganisms, among which especially *Aspergillus* sp. and *Rhizopus* sp. were engineered to synthesize diverse high-valued chemicals (Table 3).

**Table 3:** Metabolic engineering application in filamentous fungi

Category	Strategies	Strains	Products	Yield (g/g)	Titer (g/L)	Productivity (g/L/h)	Refs.
Filamentous fungi	*Aspergillus.* sp	*A. niger* iE-CexA	Citrate	–	109	0.52	[79]
		*A. oryzae* CMPIMISN-3	l-malate	0.9	117.2	1.17	[93]
		*A. terreus* HZ-ΔlovF4-lovE	Monacolin J	–	5.5	0.02	[98]
		*A. terreus* XH86-8	Itaconate	–	80	0.83	[142]
		*A. oryzae* GAAF41	l-malate	0.82	82.3	1.18	[97]
		*A. oryzae* 2103a-68.1	l-malate	1.11	66.3	1.05	[94]
		*A. niger* D15	Mannanase	–	28.91	0.17	[4]
		*A. oryzae* WS-M-P-PP-C4-MA-PFK	l-malate	–	165	1.38	[143]
		*A. niger* US368	Xylanase	–	1.87	–	[4]
		*A. niger* S1149	l-malate	1.22	201.13	1.05	[96]
		*A. niger* S575	l-malate	0.95	201.24	0.93	[96]
		*A. niger* H915-1	Itaconate	–	4.92	0.05	[144]
		*A. oryzae* CDC14(3)	l-malate	0.75	142.5	1.08	[145]
		*A. niger* iE-CexA	Citrate	–	109	0.52	[79]
		*A. oryzae* CMPIMISN-3	l-malate	0.9	117.2	1.17	[93]
	*Rhizopus.* sp	*R. oryzae* UV1	l-lactate		91.7		[102]
		*R. oryzae* WHT5	Fumarate	–	49.5	0.41	[103]
		*R. oryzae* DG-3	Fumarate		44.1		[104]
		*R. delemar* HF121	l-malate	–	120	2	[105]
		*R. oryzae*	Fumarate	0.78	–	–	[107]
		*R. oryzae* G80	Fumarate	0.314	17.1	0.192	[110]
		*R. arrhizus* RH 7-13-9#	Fumarate	–	30.3	–	[111]
		*R. oryzae*	Fumarate	–	21.9	0.23	[146]
		*R. oryzae* pLdhA71X	l-lactate	–	77.5	1.1	[147]

### Application of *Aspergillus* sp. as microbial cell factories

*Aspergillus* sp. (*A. niger*, *A. oryzae*, *A. terreus*, *A. flavus*) possess natural advantages in the production of enzymes, organic acids, and other high-value products due to their powerful hydrolytic enzyme system and strong protein secretion pathway that enable it to grow and reproduce quickly and adapt to harsh environments [77–80]. Furthermore, researchers developed strategies for more efficient production based on high protein secretion efficiency, genomic information, and low culture costs[4]. Therefore, mutation screening and metabolic engineering applications can enhance the production efficiency of *Aspergillus* sp. (Table 3)*.*

#### Mutant screening in* Aspergillus* sp.

Strategies for mutant screening mainly include high-throughput breeding, morphology screening, and adaptive laboratory evolution, which is the direct method to increase the efficiency of microbial cell factories [4, 81]. High-throughput breeding was successfully applied to enhance antifungal activity, glucosidase activity, and gluconate production by improving cellular metabolism [78, 80, 82]. For example, resazurin as used as the indicator for high-throughput screening to improve antifungal activity, which was successfully identified in 2.7% of 12,000 microbial extracts [83]. For glucosidase activity, the correlation between the HC value and FPA was used in conjunction with 24-square deep-well microliter plate fermentation. The best strain *A. niger* H11201 exhibited 38.74 and 63.23% enhancements in the filter paper assay and glucosidase activity, respectively [84]. Another example is the development of a correlation coefficient between CuSO_4_ data and HPLC data for detecting gluconate. Furthermore, the mutant IV-7-C6 exhibited a gluconate production rate of 0.077 mol/L/h, representing a 32.8% improvement compared to that of parent strain [82]. When applying morphological screening, the percentage of vegetative mycelia and specific surface area are important parameters to regulate the growth and chemical production of *A. oryzae*. For example, a high percentage of vegetative (nonconducting and white) mycelia was not conducive for l-malate production. By combining other screening parameters with three rounds of combined mutagenesis based on ARTP, ^60^Co-γ, and NTG, the mutant FMME-S-38 was selected, which exhibited an l-malate titer of 164.9 g/L, with a productivity of 1.14 g/L/h in a 30-L fermenter [85]. Another example is the improvement of the specific surface area for enhancing nutrient absorption. The mutant FMME-218-37 exhibited a specific surface area of mycelia and pellets of 6.82 μm^−1^ and 6.98 mm^−1^, representing respective improvements of 65.91% and 20.97% compared to the control. Finally, the l-malate titer and productivity reached 95.2 g/L and 0.57 g/L/h in the 7.5-L fermenter, respectively [86]. Adaptive laboratory evolution can enhance the production of target chemicals and reduce the generation of byproducts. For example, to improve cellulase production, *A. niger* was cultured in minimal medium with 1% (w/v) a-cellulose and 1.5% (w/v) agar, followed by transcriptomic analysis. Finally, the evolved strain CBS 140717 exhibited a fivefold increase of cellulase production [87]. In an example of adaptive evolution to reduce byproduct generation, *A. oryzae* 3.042 was evolved to reduce tyrosine crystals. After further fermentation optimization, the amount of tyrosine crystals was reduced to 5.67 mg/g dry material [88].

#### Metabolic engineering applications in* Aspergillus* sp.

Metabolic engineering strategies in *Aspergillus* sp. mainly include CRISPR techniques and metabolic flux regulation, which can increase the efficiency of microbial cell factories [4, 89, 90] (Table 1). CRISPR techniques alow the accurate and easy editing of chromosomal genes for enhancing pathway efficiency [91] For example, the CRISPR/Cas9 technique was developed in *A. niger* to achieve the modification of genes through single base editing. The deaminase, Cas9 nickase, and uracil glycosylase inhibitor were used to build the CRISPR/Cas9-rAPOBEC1 base editing system. Finally, the *pyrG* and *fwnA* genes were inactivated using this system with an efficiency of 47–100% [92]. Based on this, CRISPR was used to identify the transport protein CexA through loss-of-function mutations, and an engineered strain with inducible *pmbfA* gene expression showed a higher performance than the control strain with constitutive expression. Finally, 109 g/L of citric acid were obtained in the inducible system, representing a threefold improvement compared to the constitutive expression system [79].

In addition to metabolic flux regulation, studies investigated compartment engineering, pathway engineering, and optimization of substrate utilization. For example, compartment engineering can also be used in *A. oryzae* for synthesizing l-malate, and the glyoxylate bypass for l-malate was enhanced in the mitochondria to improve carbon metabolism. After the incorporation of other metabolic engineering strategies, the l-malate titer, yield, and productivity were increased to 117.2 g/L, 0.9 g/g, and 1.17 g/L/h, respectively [93]. For pathway engineering, the *pyc*, *mdh3*, and the l-malate transporter (C4T318) involved in l-malate synthesis were constitutively expressed to construct the *A. oryzae strain* 2103a-68, which exhibited a threefold increase of specific l-malate production rate to 1.87 mmol/gDCW/h [94]. To redistribute the flux toward l-malate from citrate, the transporter MstC and the key enzymes in the glycolic pathway were overexpressed to form the *A. niger* strain S1149, which produced an l-malate titer of 201.13 g/L, with a yield of 1.64 mol/mol productivity of 1.05 g/L/h [95]. Furthermore, the Cre-loxP-based genetic system was utilized to delete the *oahA* gene, as well as insert *pyc, mdh3* and C4dicarboxylate transporter gene c4t318. The resulting engineered strain S575 produced up to 201.24 g/L l-malate in a 2-L fermenter, while also producing 28 g/L citrate and 1.87 g/L fumarate [96]. For substrate utilization, the *glaA*, amylase (*amyB*), and glucosidase (*agdA*) genes were overexpressed in *A. oryzae* to utilize corn starch, while fumarase was used to promote fumarate transformation for l-malate production [97]. In addition, *A. terreus* is a potential industrial filamentous fungus for food and pharmaceutical biotechnology, which can produce itaconate and various pharmaceutical [5]. The several cryptic biosynthetic gene clusters can be analyzed by genome mining strategy and improved the synthetic capacity and fermentation capability of chemicals [98].

### Applications of *Rhizopus* sp. as microbial cell factories

*Rhizopus stolonifera*, *Rhizopus delemar*, *Rhizopus microspores*, and *Rhizopus arrhizus* have all been studied in detail, while *Rhizopus oryzae* is a typical industrial strain [99, 100]. *R. oryzae* and *R. delemar* can produce saccharifying enzymes, glucosidase, lactate, fumarate and malate using starch as a cheap substrate [101]. Mutant screening and metabolic engineering can be applied in *R. oryzae* and *R. delemar* to construct more efficient cell factories (Table 3).

#### Mutant screening in* Rhizopus* sp.

Respiration-deficient, furfural-resistant, and 2-deoxyglucose-resistant mutant can be obtained through mutant screening. To obtain respiration-deficient mutants of *R. oryzae,* ultraviolet irradiation was used to generate a mutant library of *R. oryzae* AS 3.3461, and triphenyltetrazolium chloride (TTC) upper medium was utilized to screen the respiration-deficient mutants. In the resulting strain UV-1, the ATP content was decreased by 27.6% and lactate dehydrogenase (LDH) activity was increased by 22.7%. Finally, the l-lactate titer and productivity of mutant UV-1 reached 91.7 g/L and 1.53 g/L/h, respectively [102]. A furfural-resistant mutant was obtained by using different concentrations of furfural to culture *R. oryzae* HF120. Then, corncob hydrolytes was used as substrate to culture the mutant WHT5, which produced 49.5 g/L fumarate with a productivityof 0.41 g/L/h [103]. One of the mechanisms of 2-deoxyglucose resistance is the enhancement of glycolysis activity, and corresponding mutants can exhibit higher glucose consumption and improved target product synthesis [86]. The mutant *R. oryzae* DG-3 could initially produce 39.8 g/L fumarate, which was further increased to 44.1 g/L by simultaneous saccharification and fermentation in a 3-L stirred-tank bioreactor [104]. In addition, the contaminated citric acid fermentation medium was used to isolate *R. delemar* HF121, which produced an l-malate titer of 120 g/L with a productivity of 2 g/L/h [105].

#### Metabolic engineering of* Rhizopus* sp.

Metabolic engineering applications in *R. oryzae* mainly include pathway construction and evolutionalry engineering, which can channel pathway fluxes toward target chemicals [106–108]. Heterologous expression for pathway construction have already been developed in *R. oryzae.* For example, the endogenous pyruvate carboxylase gene *pyc5* and exogenous phosphoenolpyruvate carboxylase gene *ppc1* can be expressed to improve the titer of oxaloacetate, which is the precursor of fumarate. Thile the *pyc5* transformant could only produce small amounts fumarate, the *ppc1* transformant achieved a fumarate yield to 0.78 g/g, representing a 26% improvement [107]. Evolutionary engineering, which can utilize natural selection to obtain desired traits, can increase enzyme activity and cellular synthetic ability [109]. For example, various crude glycerol concentrations were used to improve the glycerol utilization and tolerance of *R. oryzae* wild1.22. The evolved strain G80 was selected in medium with 80 g/L crude glycerol, ad reached a fumarate titer, yield and productivity of 17.1 g/L, 0.314 g/g, and 0.192 g/L/h, respectively. Finally, 25.5 g/L fumarate was obtained in 1-L shake flasks, representing a 20.9-fold increase [110]. In addition, cell immobilization can also improve fumarate production [111].

## Discussion

**Fig. 2:**
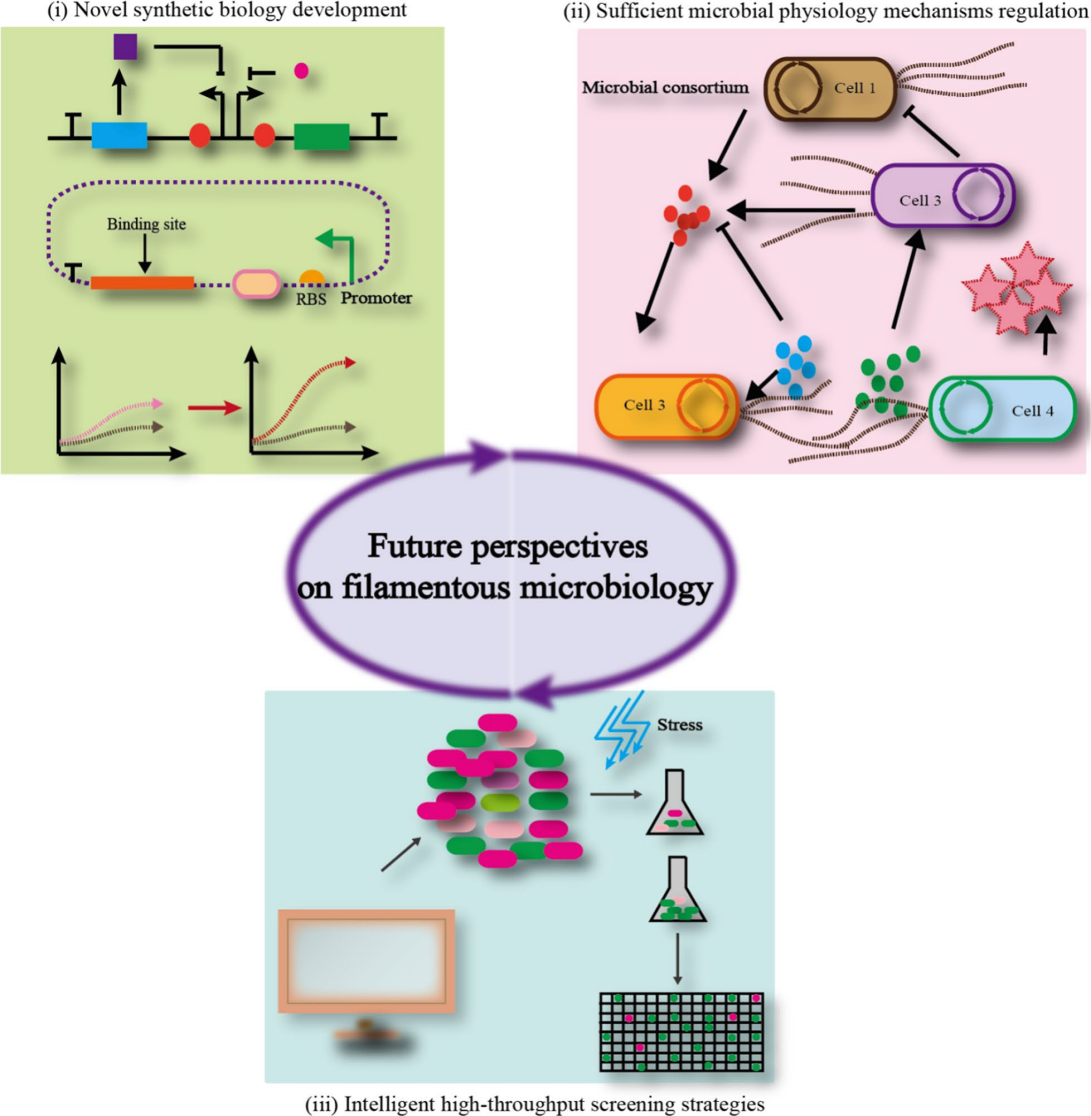
Future perspectives on Filamentous bacteria, yeast, and fungi. The novel synthetic biology development, sufficient microbial physiology mechanisms regulation, and intelligent high-throughput screening strategies could assist the development of filamentous microbiology cell factory

Filamentous bacteria, yeasts, and fungi offer an alternative chassis for synthesizing high-valued chemicals, which mainly include enzymes, organic acids, and pharmaceuticals. Current methods can not only utilize these strains for chemical products, but also can endow them with novel functions to reprogram microbial metabolism [112, 113]. Furthermore, metabolic engineering strategies and synthetic biology tools can improve the production performance, which includes the synthetic capacity, growth performance, and cellular adaptation [114, 115]. Therefore, filamentous bacteria, yeasts, and fungi can translate to the microbial industry through intelligent and artificial strategies [116, 117]. Although filamentous organisms provide many advantages to enhance the efficiency of microbial cell factories, there is still a need to study microbial metabolism based on future perspectives (Fig. 2):

(i) Novel synthetic biology developments, new metabolic engineering tools, and intelligent manipulation systems are typical strategies for improving the efficiency of microbial cell factories. The new metabolic engineering tools integrate extracellular environment signals (light, temperature, electricity, and ultrasound) and intracellular signals (biosensors, quorum sensing, genetic circuits) [118, 119]. Furthermore, a modular synthetic biology toolkit can be built for filamentous fungi microorgnaisms, which can be more rapidly assembled in a standardized and modular manner [106, 120]. Intelligent manipulations are mainly based on the new metabolic engineering tools for achieving the autonomous dynamic regulation for the synthesis of chemicals, such as N-acetylglucosamine [121], glucaric acid [122], shikimate [123], and other high-value compounds [124].
(ii) Sufficient understanding of microbial physiology, organelles, cell morphology, and artificial consortia can allow us to design artificial novel functions to regulate the pathway optimization and cell–cell communication [125–128]. For example, the sum is greater than the parts in microbial consortia, which can achieve complex functions and produce challenging chemicals [129, 130]. In addition to microbial organelles, artificial phase separation can enhance the pathway enzyme transformation and regulate cell behavior [131, 132]. Cellular morphology can be regulated in the *E. coli*, yeasts, and *Aspergillus* sp. through metabolic engineering and biochemical strategies to produce high-valued chemicals [22, 85].
(iii) Intelligent high-throughput screening strategies can be applied because filamentous bacteria, yeasts, and fungi often possess long culture time and limited rational screening tools, which limits their development into efficient cell factories [133, 134]. Therefore, the productive performance of filamentous bacteria, yeasts, and fungi should be assisted by intelligent high-throughput screening strategies, which mainly include random assembly-based strategies, microfluidics technology, high-throughput culture systems, electrochemical sensor-based screening, and biosensor-based screening [135, 136]. Furthermore, the application of big-data and artificial intelligence could revolutionize current screening tools and accelerate the translation from cell to industry [137, 138].

## Data Availability

All data generated or analyzed during this study are included in this published article and its supplementary information files.
